# Urinary proteomics for prediction of mortality in patients with type 2 diabetes and microalbuminuria

**DOI:** 10.1186/s12933-018-0697-9

**Published:** 2018-04-06

**Authors:** Gemma E. Currie, Bernt Johan von Scholten, Sheon Mary, Jose-Luis Flores Guerrero, Morten Lindhardt, Henrik Reinhard, Peter K. Jacobsen, William Mullen, Hans-Henrik Parving, Harald Mischak, Peter Rossing, Christian Delles

**Affiliations:** 1 Institute of Cardiovascular and Medical Sciences, University of Glasgow, 126 University Place, Glasgow, G12 8TA UK; 2 Steno Diabetes Center, Gentofte, Copenhagen, Denmark; 3 Mosaiques Diagnostics, Hanover, Germany; 4 HEALTH, University of Aarhus, Aarhus, Denmark; 5 Institute for Clinical Medicine, University of Copenhagen, Copenhagen, Denmark

**Keywords:** Diabetes, Microalbuminuria, Proteomics, Mortality, Biomarkers

## Abstract

**Background:**

The urinary proteomic classifier CKD273 has shown promise for prediction of progressive diabetic nephropathy (DN). Whether it is also a determinant of mortality and cardiovascular disease in patients with microalbuminuria (MA) is unknown.

**Methods:**

Urine samples were obtained from 155 patients with type 2 diabetes and confirmed microalbuminuria. Proteomic analysis was undertaken using capillary electrophoresis coupled to mass spectrometry to determine the CKD273 classifier score. A previously defined CKD273 threshold of 0.343 for identification of DN was used to categorise the cohort in Kaplan–Meier and Cox regression models with all-cause mortality as the primary endpoint. Outcomes were traced through national health registers after 6 years.

**Results:**

CKD273 correlated with urine albumin excretion rate (UAER) (r = 0.481, p = <0.001), age (r = 0.238, p = 0.003), coronary artery calcium (CAC) score (r = 0.236, p = 0.003), N-terminal pro-brain natriuretic peptide (NT-proBNP) (r = 0.190, p = 0.018) and estimated glomerular filtration rate (eGFR) (r = 0.265, p = 0.001). On multivariate analysis only UAER (β = 0.402, p < 0.001) and eGFR (β = − 0.184, p = 0.039) were statistically significant determinants of CKD273. Twenty participants died during follow-up. CKD273 was a determinant of mortality (log rank [Mantel-Cox] p = 0.004), and retained significance (p = 0.048) after adjustment for age, sex, blood pressure, NT-proBNP and CAC score in a Cox regression model.

**Conclusion:**

A multidimensional biomarker can provide information on outcomes associated with its primary diagnostic purpose. Here we demonstrate that the urinary proteomic classifier CKD273 is associated with mortality in individuals with type 2 diabetes and MA even when adjusted for other established cardiovascular and renal biomarkers.

**Electronic supplementary material:**

The online version of this article (10.1186/s12933-018-0697-9) contains supplementary material, which is available to authorized users.

## Background

It is estimated that around 35% of individuals affected by type 2 diabetes will develop DN [1] which is characterised by elevated UAER and declining renal function. Patients with DN are at particularly high risk of mortality, driven primarily by cardiovascular disease [2]. Microalbuminuria [MA, defined as UAER > 30 mg/24 h or urinary albumin creatinine ratio (UACR) 30 mg/g creatinine] is the earliest clinical hallmark of DN, and both UAER and eGFR have been shown to independently predict cardiovascular mortality as well as renal events in patients with type 2 diabetes [3]. Despite this, the utility of MA as a biomarker is limited by a number of factors including its variability and lack of sensitivity at low levels [4]. Furthermore, progression rates amongst individuals with MA are lower than previously thought [5] and we now know that MA will regress in around one-third of individuals [6]. As a result, alternative biomarkers to identify those at highest risk of progressive renal disease and associated cardiovascular diseases are needed in order to target intensified risk factor management and preventative therapies towards this subpopulation.

Proteomics involves the large-scale separation and quantification of proteins and polypeptides within a biological sample and is a promising technique for identification of complex conditions such as DN, as features derived from multiple underlying disease pathways can be measured simultaneously. Urine is an attractive biofluid for proteomic studies in the context of DN as it is not only produced by the kidneys but also contains peptides that are stable and can be subjected to mass spectrometry without further digestion. Urinary proteomic studies have demonstrated significant associations of multiple urinary peptides with diabetes [7] and DN [8]. The CKD273 classifier is a panel of 273 peptides which are differentially regulated in the urine of patients with chronic kidney disease (CKD) compared to healthy controls [9]. CKD273 has shown promise as a tool for early detection of DN risk [10–12] and this is currently being assessed in a multicentre prospective clinical trial [13]. However, the utility of CKD273 as a predictor of death associated with DN has not yet been investigated.

We have previously shown in a cohort of 200 individuals with type 2 diabetes and MA who were free from coronary artery disease at baseline that NT-proBNP and CAC score are determinants of fatal and non-fatal cardiovascular events as well as all-cause mortality over a median follow-up period of 6.1 years [14]. We have now analysed baseline samples from this cohort to study the association of CKD273 with mortality.

## Methods

### Participant recruitment

Recruitment, study procedures and sample size considerations have previously been published elsewhere [14, 15]. In brief, a cohort of 200 patients with type 2 diabetes receiving intensive multifactorial intervention as per the Steno 2 protocol [16] were identified at the Steno Diabetes Center over a 12 month period between February 2007 and February 2008. Individuals with symptoms suggestive of coronary artery disease were excluded from the study, as were those with Q-waves evident on 12-lead electrocardiography; contraindications to CT angiography including abnormal plasma creatinine; and malignancy. All participants gave informed consent; the study was approved by the local ethics committee and is in accordance with the Declaration of Helsinki.

### Study procedures

*Cardiac computed tomography* was conducted using a 16 multidetector-row CT scanner with 3 mm slice thickness during a single breath hold (Philips Precedence MX 8000IDT 16-slice; Philips Medical Systems, Best, The Netherlands). A separate workstation with dedicated software (Heartbeat-CS, EBW; Philips Medical Systems) was used to quantify total CAC score for each participant based on intimal and medial calcification in the left main, left anterior descending, circumflex and right coronary arteries. *NT*-*proBNP* was measured at baseline in all participants by immunoassay as previously described [17]. *UAER* was measured by immunoassay in three consecutive 24-h collections and geometric mean was calculated [18]. The Chronic Kidney Disease Epidemiology Collaboration (CKD-EPI) equation was used to calculate *eGFR* from plasma creatinine [19].

### Urinary proteomics

#### Sample selection and preparation

Urine samples from baseline visits were used for the present study with selection based on sample availability. Specimens were available from 188 participants however 33 of these failed quality control assessment, leaving 155 datasets for analysis. Overall, clinical characteristics of these patients were not different from the whole study group except for higher levels of UAER in samples that failed quality control for proteomics (data not shown). Specimens were stored at − 80 °C from collection until preparation. A 0.7 mL aliquot was thawed, diluted with 0.7 mL urea (2 mol/L) and NH_4_OH (10 mmol/L) containing 0.02% sodium dodecyl sulphate prior to filtration at 3000 g using a Centrisart ultracentrifugation device with molecular mass cut-off of 20 kDa (Sartorius, Göttigen, Germany) until 1.1 mL of filtrate was obtained. Samples were then desalinised using PD-10 columns (GE Healthcare, Stockholm, Sweden) equilibrated with 0.01% NH_4_OH in HPLC-grade water. Samples were lipophilised and resuspended in HPLC-grade water to a final protein concentration of 0.8 µg/µL.

#### Sample and data processing

Study team members were blinded to clinical characteristics during sample analysis. Urinary proteomic analysis was performed by capillary electrophoresis coupled to mass spectrometry (CE–MS) using a P/ACE MDQ capillary electrophoresis system (Beckman Coulter, USA) coupled to micro-time of flight mass spectrometer (Bruker Daltonic, Germany) as previously described [9]. The repeatability and stability of this technique has previously been evaluated [20]. Mass spectral ion peaks representing identical molecules at different charge states were deconvoluted into single masses using bespoke Mosaiques Visu software [21]. Normalisation of analytical and urine dilution variances was performed against 29 “housekeeper” peptides which are consistently present in urine with minimal relative standard deviation (SD) [22]. All detected peptides were deposited, matched and annotated in a Microsoft SQL database. Data pertaining to the 273 urinary peptides represented by the CKD273 classifier were then translated into a single numerical score, or classification factor, using support vector machine modelling. Previous studies have confirmed that a score of > 0.343 is the best established threshold for identification of patients at highest risk of progression to overt DN [9, 10, 12]. Additional analyses were performed with the CAD238 urinary proteomic classifier, generated using the same methodology, for detection of coronary artery disease [23].

### Endpoints

The primary endpoint was defined as death from any cause. Secondary endpoints were cardiovascular events (4-point MACE including non-fatal myocardial infarction, stroke, hospitalisation for heart failure and cardiovascular death); decline in eGFR of at least 30% and transition to macroalbuminuria at any time point during the follow up period. All study participants were traced through Danish National Health registries from 1st January 2014.

### Statistical analysis

SPSS statistics 22 (IBM Analytics, New York, USA) was used for statistical analysis. Normally distributed data are expressed as mean ± SD while nonparametric data are expressed as median (range). UAER data are expressed as geometric mean and interquartile range. Correlations were determined by Pearson’s method and high and low-risk groups were compared by 2-sample t test, both using log transformed data where appropriate. Kaplan–Meier analysis was used for survival studies where p values were derived from Log Rank (Mantel Cox) test. Cox regression analysis was performed to generate fully adjusted survival data. Receiver Operator Curve (ROC) analysis we performed on significant predictors of mortality where predicted probabilities from logistic regression analysis of individual variables were used as test variable where indicated. For comparison of areas under the the ROC curves (AUCs) the DeLong method was used in MedCalc software version 17.9.7 (Ostend, Belgium) Level of significance was set to 0.05.

## Results

### Cross-sectional analysis

Baseline characteristics of the 155 study participants in whom CKD273 classifier scores were available are shown in Table 1. With the exception of body mass index (BMI) traditional risk factors were well-controlled in the context of a multifactorial intervention strategy. Renal function was preserved (mean eGFR > 60 mL/min/1.73 m^2^) and geometric mean of UAER was within the microalbuminuric range.

**Table 1:** Baseline characteristics

Parameter	Value
Age (years)	61 (29–71)
Diabetes duration (years)	13 (1–36)
Sex (m/f)	118/37
Smokers (year/n)	42/113
BMI (kg/m^2^)	31.6 (21.6–55.6)
SBP (mmHg)	129 ± 16
DBP (mmHg)	74 ± 11
HbA1c [%] (mmol/mol)	[7.6] 58.5 (39–123)
Cholesterol (mmol/L)	3.8 (2–6.7)
HDL (mmol/L)	1.1 (0.4–3.1)
LDL (mmol/L)	1.7 (0.4–4.4)
Creatinine (µmol/L)	77 ± 17.6
eGFR (mL/min/1.73 m^2^)	88 ± 17
UAER (mg/24 h)	85 [34;194]
CKD273	0.268 (− 1.08 to 1.23)
CAD238	− 0.513 ± 0.256
NT-proBNP (ng/L)	45 (5–576)
CAC score	501 ± 771

*BMI* body mass index, *SBP* systolic blood pressure, *DBP* diastolic blood pressure, *HbA1c* glycated haemoglobin, *HDL* high density lipoprotein, *LDL* low density lipoprotein, *eGFR* estimated glomerular filtration rate, *UAER* urine albumin excretion rate, *NT-proBNP* N-terminal pro-brain natriuretic peptide, *CAC* coronary artery calcium. Data are mean ± SD or median (range). UAER expressed as geometric mean and interquartile range. eGFR determined by CKD-EPI formula

Examination of the dataset according to CKD273 classifier score revealed that 69 participants (45%) had CKD273 classifier score > 0.343, while 86 (55%) fell below this threshold. Comparison of clinical and biochemical parameters according to CKD classifier risk threshold is shown in Table 2. While age, BMI, blood pressure, glycated haemoglobin (HbA1c) were similar between high and low risk patients, those with classifier score above 0.343 had lower eGFR and higher UAER. In addition, these participants also had significantly higher CAC score.

**Table 2:** Clinical characteristics according to CKD273 risk threshold

Parameter	CKD273 > 0.343 (n = 69)	CKD273 < 0.343 (n = 86)	p value
Age (years)	62 (32–71)	61 (29–71)	0.173
Diabetes duration (years)	13 (1–35)	12.5 (1–36)	0.455
Gender (m/f)	59/10	59/27	*0.014
Smokers (year/n)	24/45	18/68	0.054
BMI (kg/m^2^)	31 (23–56)	32 (22–48)	0.324
SBP (mmHg)	130 ± 17	129 ± 16	0.725
DBP (mmHg)	74 ± 11	75 ± 12	0.574
HbA1c [%] (mmol/mol)	[7.5] 58 (42–110)	[7.6] 60 (39–123)	0.267
Cholesterol (mmol/L)	3.9 (2.3–6.7)	3.8 (2–6.6)	0.893
HDL (mmol/L)	1.1 (0.4–2.2)	1.1 (0.7–3.1)	0.066
LDL (mmol/L)	1.8 (0.5–4.4)	1.7 (0.4–4.2)	0.342
Creat (µmol/L)	80 ± 18	75 ± 17	*0.049
eGFR (mL/min/1.73 m^2^)	86 ± 18	90 ± 15	0.143
UAER (mg/24 h)	148 [70;385]	55 [29;99]	*< 0.001
CKD273	0.546 (0.369–1.231)	0.041 (− 1.078 to 0.347)	*< 0.001
CAD238	− 0.463 ± 0.202	− 0.586 ± 0.281	*0.002
NT-proBNP (ng/L)	52 (5–550)	42 (5–576)	0.615
CAC score	676 ± 961	363 ± 547	0.018

*BMI* body mass index, *SBP* systolic blood pressure, *DBP* diastolic blood pressure, *HbA1c* glycated haemoglobin, *HDL* high density lipoprotein, *LDL* low density lipoprotein, *eGFR* estimated glomerular filtration rate, *UAER* urine albumin excretion rate, *NT-proBNP* N-terminal pro-brain natriuretic peptide, *CAC* coronary artery calcium. Data are mean ± SD or median (range). UAER expressed as geometric mean and interquartile range. eGFR determined by CKD-EPI formula. p < 0.05 and * denote statistical significance. Comparisons between groups are by 2-sample t test or appropriately transformed data where necessary. Numerical CKD273 cut-off for diagnosis of DN is 0.343

We then analysed CKD273 classifier score as continuous variable in the whole study group and found an inverse correlation with eGFR (r = − 0.265, p = 0.001) and a direct correlation with UAER (r = 0.481, p = <0.001). There was no significant difference in CKD273 between male and female participants, and no correlation with other clinical parameters with the exception of age (Additional file 1: Figure S1). In a multivariate regression model including age, sex, eGFR and UAER; only eGFR and UAER remained as significant determinants of CKD273 classifier score, together explaining 30% of its variability. Pearson correlation analysis did reveal a linear association between CKD273 score and the cardiovascular biomarkers NT-proBNP and CAC score (Additional file 1: Figure S2).

### Longitudinal analysis

During the follow up period 19 participants (12%) transitioned from micro- to macro-albuminuria, while 29 (18%) experienced a 30% decline in eGFR. Eight participants (5%) experienced both endpoints. Baseline CKD273 score was higher in those who experienced a renal endpoint compared to those who did not, but only reached statistical significance in those who transitioned in albuminuria status (Table 3). Kaplan–Meier analysis showed that CKD273 score above the risk threshold for DN was not predictive of a 30% eGFR decline over a 6-year follow up period in this cohort (Additional file 1: Figure S3), nor was classifier score correlated with absolute change in UAER (Additional file 1: Figure S4).

**Table 3:** Comparison of classifier scores in patients who reached an endpoint compared to those who did not

Endpoint	Yes	No	p value
30% decline eGFR (29 events)	0.239 ± 0.414	0.228 ± 0.390	0.886
Transition micro to macroalbuminuria (19 events)	0.354 ± 0.238	0.145 ± 0.406	*0.036
Cardiovascular event (31 events)	0.331 ± 0.389	0.216 ± 0.405	0.156
Mortality (20 events)	0.438 ± 0.355	0.208 ± 0.403	*0.017

We defined the following endpoints for analysis: (1) All-cause mortality. (2) CV events. (3) 30% decline in eGFR. (4) Transition from micro to macroalbuminuria. With regard to the latter, there were 19 patients who progressed and 73 who remained microalbuminuric or regressed; we did not analyse data on people who were macroalbuminuric at baseline. Data are mean ± SD, p value of < 0.05 deemed statistically significant. Comparisons are by 2 sample t test. p < 0.05 and * denote statistical significance

Cardiovascular events occurred in 31 (20%) participants. Baseline CKD273 classifier score was not significantly different in those who experienced a cardiovascular event, nor was it found to be associated with cardiovascular events on Kaplan–Meier analysis. Survival analysis was also performed using the CAD238 classifier, developed for prediction of cardiovascular events. Here the association with cardiovascular events during follow-up approached statistical significance and logistic regression analysis confirmed that CAD238 was associated with imaging-proven coronary artery disease whilst CKD273 was not (Additional file 1: Figure S5, Table S1).

**Fig. 1:**
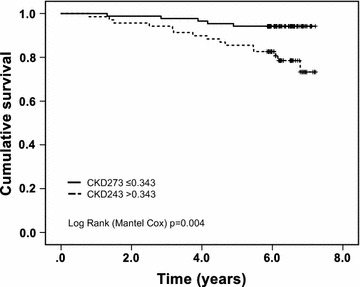
Kaplan–Meier plot CKD273 and mortality. Kaplan Meier analysis revealed that CKD273 classifier score above the predefined threshold for diagnosis of DN (0.343) was predictive of mortality over 6 years follow up (Log Rank [Mantel Cox] p = 0.004). Black line represents classifier score below 0.343; dotted line represents classifier score above 0.343

Twenty (13%) participants died during follow up. Ten (50%) of these events were cardiovascular deaths. Baseline CKD273 classifier score was significantly higher in those who died compare to survivors (Table 3). Survivors were younger and less likely to smoke (Additional file 1: Table S2). Kaplan–Meier analysis revealed that CKD273 classifier score above 0.343 was associated with mortality over 6 years follow up (Fig. 1). Active smoking was also a statistically significant determinant of death in Kaplan–Meier analysis (Log Rank (Mantel-Cox) p = 0.014). In order to adjust for continuous variables and other potential determinants of mortality we then included CKD273 (above and below the 0.343 threshold) in a more comprehensive model adjusted for age, sex, systolic blood pressure, smoking status, eGFR, UAER, CAC score and NT-proBNP. Only NT-proBNP (p = 0.03), CAC score (p = 0.006) and CKD273 (p = 0.048) remained significant determinants of mortality (supplementary Table 3). On ROC analysis the AUC of the combined predictive value of NT-proBNP and CAC score was 0.797. If this was combined with CKD273 the AUC was 0.818 (p = 0.376 for comparison of the AUCs) whereas CKD273 on its own resulted in an AUC of 0.662 (Additional file 1: Figure S6).

## Discussion

Patients with even the early stages of DN are at increased risk of death both directly and indirectly as a consequence of the associated cardiovascular disease burden [24, 25]. The presence of MA is currently the best clinically applied marker of DN risk, but it is highly variable and does not necessarily herald the onset of irreversible decline in kidney function [5, 6]. Alternative biomarkers to identify those at highest risk of comorbidity associated with diabetes would allow targeting of preventative strategies towards these individuals. Here we have shown that the CKD273 urinary proteomic classifier for detection of DN is associated with later mortality in patients with type 2 diabetes and MA.

Both UAER and eGFR are independent and additive predictors of cardiovascular events and mortality in patients with diabetes as well as within the general population [26]. In addition to these risk markers the participants in this study had baseline measurements of CAC score and NT-proBNP which have already been shown to be associated with cardiovascular events and mortality in this population [14]. CAC score, as a reflection of underlying atherosclerotic disease burden, has been shown in several studies to reliably inform assessment of all-cause mortality and cardiovascular event risk in individuals with diabetes [27–29]. NT-proBNP is secreted in response to cardiomyocyte stretch and has also been shown to powerfully predict mortality in cohorts of individuals with diabetes [17, 30]. The availability of these parameters offered us the opportunity to determine the performance of the CKD273 classifier in comparison to already established clinical tools with proven value in predicting mortality. Our data suggest that the CKD273 is associated with mortality in this cohort of patients with type 2 diabetes and MA independently of these established risk markers.

The CKD273 urinary proteomic classifier has shown some promise as a tool for detection of DN risk. Classifier score has been demonstrated to be associated with later progression to MA in normoalbuminuric patients [10, 12]; to identify individuals who later transition to macroalbuminuria [11]; and to change towards a “healthier” value in patients treated with renin angiotensin system blocking agents [31]. The utility of CKD273 in the early detection of DN followed by specific treatment is currently being formally tested prospectively in a multicentre trial [12]. Studies in populations with CKD of mixed aetiology have shown that CKD273 score is predictive of later requirement for dialysis [32] and is useful for identification of those who display rapid decline in eGFR during follow-up [21]. It seems surprising then that we have not been able to show the same potential in this cohort. This may simply be an issue of statistical power as the number of renal endpoints was small and follow up relatively short, however it should also be considered in the context of recent general population data which suggested that the CKD273 classifier is most useful as a predictor of progressive kidney disease in patients with preserved renal function (eGFR > 70 mL/min/1.73 m^2^) and is of less value in cohorts where renal disease is already established [33, 34]. The mean eGFR in our cohort was 88 mL/min/1.73 m^2^ but these patients have MA, which reflects the 3rd step in the traditional 5-stage natural history of DN [35]. Given that the classifier shows most promise as an early, in fact “pre-clinical”, predictor of progressive DN we would not necessarily expect it to perform as well in a cohort of patients where the disease is already established.

The main finding of this study is that the CKD273 classifier was associated with all-cause mortality in our cohort and so could provide additional information on other comorbidities associated with CKD. CKD273 is a composite of 273 differentially regulated urinary peptides. These are primarily fragments of collagens type 1 and type 3 but peptides corresponding to source proteins such as albumin, uromodulin and α-1 antitrypsin are also captured by the classifier, which therefore simultaneously reflects multiple altered disease pathways [9]. In view of the fact that altered collagen turnover is key to progression of both kidney disease and cardiovascular disease and collagens type 1 and 3 are more predominant within the vasculature than the glomerular basement membrane it seems intuitive that CKD273 may not only be a marker of renal but also a non-specific marker of cardiovascular disease akin to MA. Our data do not demonstrate prediction of cardiovascular events by CKD273 in patients with type 2 diabetes and MA, however the association of CKD273 with DN and altered collagen turnover do suggest a link to microvascular disease and hence mortality. In recent years specific proteomic panels have been developed for identification of atherosclerotic disease in animal models and for prediction of CAD in human subjects [36, 37]. The CAD238 classifier for prediction of coronary artery disease showed a stronger signal in this analysis and these data support the use of multimarker proteomic panels for diagnosis of specific conditions.

This is the first study to test the predictive power of the CKD273 classifier for all-cause mortality in a cohort of patients with MA and preserved eGFR in comparison to other established risk markers such as NT-proBNP and CAC score. Strengths of this study include a well-phenotyped cohort with longitudinal follow-up data available. In addition we were able to formally test the predictive power of the CKD273 classifier alongside a number of established clinical risk markers such as eGFR, UAER, CAC score and NT-proBNP, which is a prerequisite when determining the utility of novel biomarkers. There are however several limitations to consider. Firstly the original sample size is relatively small and event rate over the 6-year follow up period is low, both of which may have affected the robustness of our analysis. In addition, although analysed as a secondary outcome, our choice of 30% eGFR decline as a renal endpoint is less robust than doubling of serum creatinine or 40% eGFR decline. This decision was made based on the fact that the number of renal endpoints was low and the primary aim of this analysis was to assess prediction of mortality rather than renal events. Although our data suggest association between CKD273 and mortality independent of a selection of established traditional and novel risk biomarkers it should be borne in mind that multiple processes, including for example inflammation and oxidative stress, underpin the development of cardiovascular and renal disease in diabetes [38] [39, 40]. Whilst multimarker omics strategies offer the opportunity to capture changes in multiple disease processes simultaneously numerous alternative biomarkers remain under evaluation and for the time being few have made the transition into routine clinical practice.

## Conclusions

In conclusion, the CKD273 urinary proteomic classifier is associated with mortality in this cohort of patients with type 2 diabetes and MA independent of selected established clinical risk markers. These findings, although promising, require further confirmation on a larger scale in independent cohorts.

## Additional file

**Additional file 1: Figure S1.** Univariate determinants of CKD273. eGFR, estimated glomerular filtration rate; UAER, urine albumin excretion rate. Analysis by Pearson correlation on appropriately transformed data where necessary. CKD273 classifier score correlated with age (panel A; r=0.238, p=0.003); eGFR (panel B; r=-0.265, p=0.001) and UAER (panel C; r=0.481, p=<0.001). There was no significant difference in classifier score between men and women, and no correlation with other traditional clinical parameters. **Figure S2.** Correlations between CKD273 and cardiovascular biomarkers. CAC, coronary artery calcium score; BNP, brain natriuretic peptide. CKD273 correlated with CAC score (left panel; r=0.236, p=0.003) and NT-proBNP (right panel; r=0.190, p=0.018). Correlations by Pearson’s method on appropriately transformed data where necessary. P < 0.05 deemed statistically significant. **Figure S3.** Kaplan Meier plot of CKD273 and primary renal endpoint (30% decline in eGFR). eGFR, estimated glomerular filtration rate. Blue line represents classifier score < 0.343; green line represents classifier score > 0.343. Kaplan Meier analysis revealed that CKD273 classifier score above the predefined threshold for diagnosis of DN (0.343) did not predict 30% decline in eGFR (Log Rank [Mantel Cox] p=0.598). **Figure S4.** Correlation plot showing relationship between CKD273 and change in UAER over follow up. Correlation is by Pearson’s method on log10 transformed data. P < 0.05 deemed statistically significant. The correlation between CKD273 classifier score at baseline and change in UAER did not reach statistical significance (r=0.249, p=0.072). **Figure S5.** Kaplan Meier plot of CAD238 and cardiovascular events over follow up. Kaplan Meier analysis revealed that CAD238 classifier score in the highest quartile showed a trend towards prediction of cardiovascular events which did not reach statistical significance (Log Rank [Mantel Cox] p=0.055). **Table S1.** Logistic regression model for imaging-proven CAD. Here we use NT-proBNP and coronary artery calcification scores above thresholds determined in a previous publication for identification of significant CAD. CKD273 is not associated with imaging-proven CAD in this analysis. NT-proBNP, N-terminal pro-brain natriuretic peptide. **Table S2.** Baseline characteristics in participants who died during follow-up and survivors. BMI, body mass index; SBP, systolic blood pressure; DBP, diastolic blood pressure; HbA1c, glycated haemoglobin; HDL, high density lipoprotein; LDL, low density lipoprotein; eGFR, estimated glomerular filtration rate; UAER, urine albumin excretion rate; NT-proBNP, N-terminal pro-brain natriuretic peptide; CAC, coronary artery calcium. Data are mean ± SD or median (range). UAER expressed as geometric mean and interquartile range. eGFR determined by CKD-EPI formula. **Table S3.** Fully adjusted Cox regression model for total mortality. UAER, urinary albumin excretion rate; eGFR, estimated glomerular filtration rate. **Figure S6.** Receiver operator characteristic (ROC) analysis. The following variables were tested for the outcome “mortality”: CKD273 (red curve; area under the curve (AUC) 0.662); CAC score and NT-pro-BNP combined (green curve; AUC 0.797); CKD273, CAC score and NT-proBNP combined (blue curve; AUC 0.818).

## Abbreviations

AUC: area under the receiver operating curve
BMI: body mass index
CAC: coronary artery calcification
CE–MS: capillary electrophoresis coupled to mass spectrometry
CKD: chronic kidney disease
CKD-EPI: chronic kidney disease epidemiology collaboration
DN: diabetic nephropathy
eGFR: estimated glomerular filtration rate
MA: microalbuminuria
NT-proBNP: N-terminal pro brain natriuretic peptide
ROC: receiver operating curve
UAER: urine albumin excretion rate
