# Supplementary material for: Urinary proteomics for prediction of mortality in patients with type 2 diabetes and microalbuminuria
Source: Cardiovasc Diabetol. 2018 Apr 6;17:50. doi: 10.1186/s12933-018-0697-9 (PMC5889591; doi:10.1186/s12933-018-0697-9)
Supplement: Supplementary file 1 — Additional file 1: Figure S1. Univariate determinants of CKD273. eGFR, estimated glomerular filtration rate; UAER, urine albumin excretion rate. Analysis by Pearson correlation on appropriately transformed data where necessary. CKD273 classifier score correlated with age (panel A; r=0.238, p=0.003); eGFR (panel B; r=-0.265, p=0.001) and UAER (panel C; r=0.481, p=<0.001). There was no significant difference in classifier score between men and women, and no correlation with other traditional clinical parameters. Figure S2. Correlations between CKD273 and cardiovascular biomarkers. CAC, coronary artery calcium score; BNP, brain natriuretic peptide. CKD273 correlated with CAC score (left panel; r=0.236, p=0.003) and NT-proBNP (right panel; r=0.190, p=0.018). Correlations by Pearson’s method on appropriately transformed data where necessary. P < 0.05 deemed statistically significant. Figure S3. Kaplan Meier plot of CKD273 and primary renal endpoint (30% decline in eGFR). eGFR, estimated glomerular filtration rate. Blue line represents classifier score < 0.343; green line represents classifier score > 0.343. Kaplan Meier analysis revealed that CKD273 classifier score above the predefined threshold for diagnosis of DN (0.343) did not predict 30% decline in eGFR (Log Rank [Mantel Cox] p=0.598). Figure S4. Correlation plot showing relationship between CKD273 and change in UAER over follow up. Correlation is by Pearson’s method on log10 transformed data. P < 0.05 deemed statistically significant. The correlation between CKD273 classifier score at baseline and change in UAER did not reach statistical significance (r=0.249, p=0.072). Figure S5. Kaplan Meier plot of CAD238 and cardiovascular events over follow up. Kaplan Meier analysis revealed that CAD238 classifier score in the highest quartile showed a trend towards prediction of cardiovascular events which did not reach statistical significance (Log Rank [Mantel Cox] p=0.055). Table S1. Logistic regression model for imaging- [file 12933_2018_697_MOESM1_ESM.docx]

**Additional data**

**Figure S1. Univariate determinants of CKD273**. eGFR, estimated glomerular filtration rate; UAER, urine albumin excretion rate. Analysis by Pearson correlation on appropriately transformed data where necessary. CKD273 classifier score correlated with age (panel A; r=0.238, p=0.003); eGFR (panel B; r=-0.265, p=0.001) and UAER (panel C; r=0.481, p=<0.001). There was no significant difference in classifier score between men and women, and no correlation with other traditional clinical parameters.

**Figure S2. Correlations between CKD273 and cardiovascular biomarkers.** CAC, coronary artery calcium score; BNP, brain natriuretic peptide. CKD273 correlated with CAC score (left panel; r=0.236, p=0.003) and NT-proBNP (right panel; r=0.190, p=0.018). Correlations by Pearson’s method on appropriately transformed data where necessary. P < 0.05 deemed statistically significant.

**Figure S3. Kaplan Meier plot of CKD273 and primary renal endpoint** (30% decline in eGFR). eGFR, estimated glomerular filtration rate. Blue line represents classifier score < 0.343; green line represents classifier score > 0.343. Kaplan Meier analysis revealed that CKD273 classifier score above the predefined threshold for diagnosis of DN (0.343) did not predict 30% decline in eGFR (Log Rank [Mantel Cox] p=0.598).

**Figure S4. Correlation plot showing relationship between CKD273 and change in UAER over follow up.** Correlation is by Pearson’s method on log10 transformed data. P < 0.05 deemed statistically significant. The correlation between CKD273 classifier score at baseline and change in UAER did not reach statistical significance (r=0.249, p=0.072).

**Figure S5. Kaplan Meier plot of CAD238 and cardiovascular events over follow up.** Kaplan Meier analysis revealed that CAD238 classifier score in the highest quartile showed a trend towards prediction of cardiovascular events which did not reach statistical significance (Log Rank [Mantel Cox] p=0.055).

|  | P-value |
| --- | --- |
| **Variables in the equation** |  |
| CAD238 | 0.010 |
| NT-proBNP > 45.2 | <0.001 |
| Coronary artery calcification score > 400 | <0.001 |
| **Variables not in the equation** |  |
| CKD273 | 0.596 |

**Table S1. Logistic regression model for imaging-proven CAD.**

Here we use NT-proBNP and coronary artery calcification scores above thresholds determined in a previous publication for identification of significant CAD. CKD273 is not associated with imaging-proven CAD in this analysis. NT-proBNP, N-terminal pro-brain natriuretic peptide.

| **Parameter** | **Non-survivors n=20** | **Survivors n=135** | **p-value** |
| --- | --- | --- | --- |
| **Age (yrs)** | 67 (50-72) | 60 (29-71) | <0.001 |
| **Diabetes duration (yrs)** | 16 (4-36) | 12 (1-35) | 0.083 |
| **Sex (m/f)** | 2/18 | 35/100 | 0.095 |
| **Smokers (y/n)** | 10/10 | 103/32 | 0.017 |
| **BMI (kg/m²)** | 30.9 (24.2-44.0) | 31.8 (21.6-55.6) | 0.717 |
| **SBP (mmHg)** | 131±19 | 129±16 | 0.598 |
| **DBP (mmHg)** | 74 (48-101) | 69 (49-92) | 0.093 |
| **HbA_1_c (mmol/mol)** | 60 (39-123) | 56 (43-91) | 0.209 |
| **Cholesterol (mmol/L)** | 4.0 (2.4-6.7) | 3.8 (2.0-6.6) | 0.989 |
| **HDL (mmol/L)** | 1.0 (0.4-2.2) | 1.1 (0.6-3.1) | 0.212 |
| **LDL (mmol/L)** | 1.7 (1.0-4.4) | 1.8 (0.4-4.2) | 0.685 |
| **Creatinine (µmol/L)** | 79±14 | 77±18 | 0.567 |
| **eGFR (mL/min/1.73m²)** | 85±16 | 89±18 | 0.278 |
| **UAER (mg/24hrs)** | 147 [47-513] | 78 [33-170] | 0.046 |
| **CKD273** | 0.487 (-.0229 – 1.231) | 0.241 (-1.078 – 1.133) | 0.020 |
| **CAD238** | -0.480±0.173 | -0.539±0.265 | 0.335 |
| **NT-proBNP (ng/L)** | 90 (5-576) | 42 (5-489) | 0.002 |
| **CAC score** | 1119±951 | 409±699 | 0.004 |

**Table S2. Baseline characteristics in participants who died during follow-up and survivors.** BMI, body mass index; SBP, systolic blood pressure; DBP, diastolic blood pressure; HbA1c, glycated haemoglobin; HDL, high density lipoprotein; LDL, low density lipoprotein; eGFR, estimated glomerular filtration rate; UAER, urine albumin excretion rate; NT-proBNP, N-terminal pro-brain natriuretic peptide; CAC, coronary artery calcium. Data are mean ± SD or median (range). UAER expressed as geometric mean and interquartile range. eGFR determined by CKD-EPI formula.

|  | P-value |
| --- | --- |
| **Variables in the equation** |  |
| CKD273 (above/below 0.343) | 0.048 |
| log10 (NT-proBNP) | 0.004 |
| Coronary artery calcification score | 0.007 |
| **Variables not in the equation** |  |
| Age | 0.174 |
| Sex (m/f) | 0.534 |
| Smoking (yes/no) | 0.175 |
| Systolic blood pressure | 0.628 |
| log10 (UAER) | 0.585 |
| eGFR | 0.569 |

**Table S3. Fully adjusted Cox regression model for total mortality.** UAER, urinary albumin excretion rate; eGFR, estimated glomerular filtration rate.

**Figure S6. Receiver operator characteristic (ROC) analysis.** The following variables were tested for the outcome "mortality": CKD273 (red curve; area under the curve (AUC) 0.662); CAC score and NT-pro-BNP combined (green curve; AUC 0.797); CKD273, CAC score and NT-proBNP combined (blue curve; AUC 0.818).
